# MicroRNA-145 Protects Cardiomyocytes against Hydrogen Peroxide (H_2_O_2_)-Induced Apoptosis through Targeting the Mitochondria Apoptotic Pathway

**DOI:** 10.1371/journal.pone.0044907

**Published:** 2012-09-18

**Authors:** Ruotian Li, Guijun Yan, Qiaoling Li, Haixiang Sun, Yali Hu, Jianxin Sun, Biao Xu

**Affiliations:** 1 Department of Cardiology, The Affiliated Drum Tower Hospital of Nanjing University Medical School, Nanjing, People’s Republic of China; 2 Center for Translational Medicine, Thomas Jefferson University, Philadelphia, Pennslyvania, The United States of America; Virginia Commonwealth University Medical Center, United States of America

## Abstract

MicroRNAs, a class of small and non-encoding RNAs that transcriptionally or post-transcriptionally modulate the expression of their target genes, has been implicated as critical regulatory molecules in many cardiovascular diseases, including ischemia/reperfusion induced cardiac injury. Here, we report microRNA-145, a tumor suppressor miRNA, can protect cardiomyocytes from hydrogen peroxide (H_2_O_2_)-induced apoptosis through targeting the mitochondrial pathway. Quantitative real-time PCR (qPCR) demonstrated that the expression of miR-145 in either ischemia/reperfused mice myocardial tissues or H_2_O_2_-treated neonatal rat ventricle myocytes (NRVMs) was markedly down-regulated. Over-expression of miR-145 significantly inhibited the H_2_O_2_-induced cellular apoptosis, ROS production, mitochondrial structure disruption as well as the activation of key signaling proteins in mitochondrial apoptotic pathway. These protective effects of miR-145 were abrogated by over-expression of Bnip3, an initiation factor of the mitochondrial apoptotic pathway in cardiomyocytes. Finally, we utilized both luciferase reporter assay and western blot analysis to identify Bnip3 as a direct target of miR-145. Our results suggest miR-145 plays an important role in regulating mitochondrial apoptotic pathway in heart challenged with oxidative stress. MiR-145 may represent a potential therapeutic target for treatment of oxidative stress-associated cardiovascular diseases, such as myocardial ischemia/reperfusion injury.

## Introduction

Myocardial infarction is one of the leading causes of morbidity and mortality worldwide. While the development of cardiac intervention techniques enables many patients to restore myocardial perfusion, the subsequent ischemia-reperfusion injury may still cause extensive cardiomyocyte death and acute heart dysfunction [1]. To date, it has been widely accepted that cardiomyocyte death under stress conditions is to a great extent a result of the activation of the mitochondrial apoptotic pathway [2]. During ischemia-reperfusion injury, cardiomyocytes typically undergo oxidative stress mediated by reactive oxygen species (ROS). ROS activates the mitochondrial apoptotic pathway (also named intrinsic apoptotic pathway), which is featured by the alteration of permeability of mitochondrial membrane and the release of cytochrome C into cytoplasm to activate the caspase cascade which ultimately lead to the degradation of genome DNA [3]–[4]. Despite of the essential role of the mitochondrial pathway in oxidative stress-mediated cardiomyocyte apoptosis, how this pathway is regulated at different levels remains elusive. MiRNA, a class of small, non-encoding RNAs that transcriptionally or post-transcriptionally modulate the expression of their target genes, has been implicated as regulatory molecules in many cardiovascular diseases, including myocardial ischemia-reperfusion injury [5]–[6]. MiR-145 is a tumor suppressor miRNA that has been recently implicated in the regulation of apoptosis networks in tumor cells through its ability of targeting various anti-apoptotic molecules [7]–[9]. Moreover, the aberrant expression of miR-145 has been shown to be associated with vascular smooth muscle cells’ response to hydrogen peroxide (H_2_O_2_)-induced oxidative stress, indicating that miR-145 may participate in the regulation of the oxidative stress-triggered apoptosis and the regulation of the mitochondrial apoptotic pathway [10]. However, little is known about whether miR-145 is associated with cardiomyocyte apoptosis under oxidative stress or how it interferes with the mitochondrial apoptotic pathway.

Accumulating evidence indicates that BH3-only proteins such as Bcl2/adenovirus E1B 19 kDa-interacting protein 3 (Bnip3) play pivotal roles in the initiation of cardiomyocyte apoptosis, necrosis, and autophagy [11]. Bnip3, which primarily localizes in the outer membrane of mitochondria, functions not only as a sensor of mitochondria to the oxidative stress in cytoplasm, but also as an effector of the mitochondria-mediated apoptosis. Bnip3 transduces the apoptotic signals through activating pro-apoptotic Bax/Bak proteins, neutralizing the anti-apoptotic BH1–4 proteins, and promoting mitochondrial membrane depolarization by inducing the formation of the pathological mitochondrial permeability transition pore (mPTP) [11]–[12]. Indeed, Bnip3 has been shown to be activated during apoptosis induced by multiple stimuli including hypoxia and ischemia-reperfusion. In vitro enforcement of Bnip3 expression promoted the cardiomyocyte apoptosis in a caspase3-independent manner [13]–[15]. Targeting the expression, localization, or activation of Bnip3 has thus been considered as a potential strategy to salvage the ischemia-reperfusion-associated cardiomyocytes apoptosis [14] [16]–[17].

Herein, we investigated the role of miR-145 in regulating cardiomyocyte apoptosis and modulating the mitochondrial apoptotic pathway in the setting of oxidative stress. Our results demonstrated that miR-145 is substantially down-regulated in cardiomyocytes with oxidative stress, and that over-expression of miR-145 significantly inhibited the H_2_O_2_-induced cellular apoptosis, ROS production, mitochondrial structure disruption as well as the activation of key signaling proteins in mitochondrial apoptotic pathway. And these protective effects were at least partially through directly targeting Bnip3.

## Materials and Methods

### Mice

Animal experiments were performed in 8–12-week-old male C57 mice, which were purchased from the Animal Center of Yangzhou University. The protocol was approved by the Institutional Animal Care and Use Committee of the affiliated Drum Tower Hospital of Nanjing University Medical School.

### Animal Model of Ischemia/reperfusion (I/R) Injury

Forty male C57 mice were randomly divided into four groups: sham 1 h, I/R 1 h, sham 3 h and I/R 3 h. Each group contains 10 mice. Surgical procedures were performed essentially as previously described [18]. Briefly, for I/R 1 h group and I/R 3 h group, mice were subjected to 50 min myocardial ischemia via ligation of the mid sections of left anterior descending arteries followed by 1 and 3 h reperfusion respectively. For sham 1 h group and sham 3 h group, animals were subjected to the procedure of opening chest followed by suture at 1 h and 3 h later respectively. The myocardial tissues bordering the primary infarction zones with approximately equal sizes were collected and stored in liquid nitrogen.

**Figure 1:**
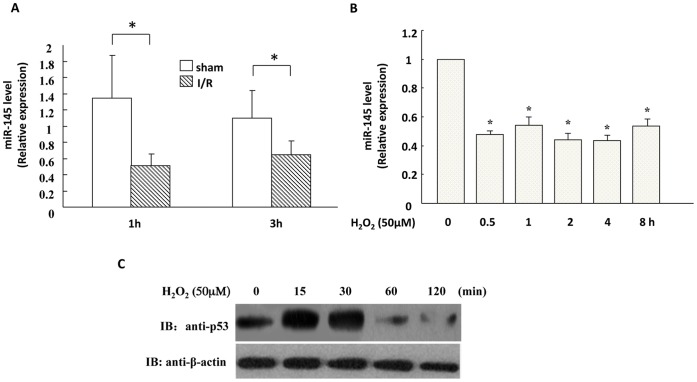
MiR-145 expression was down-regulated in ischemia/reperfused heart and H_2_O_2_-treated NRVMs. (A), Quantitative real-time PCR showed that miR-145 expression in ischemic myocardium tissues undergoing 1 hour and 3 hours reperfusion was down-regulated, compared with that in the respective sham groups. (n = 10 in each group). (B), Primary neonatal rat ventricle myocytes (NRVM) were treated with 50 µM H_2_O_2_ for indicated time courses ranging from 0.5 hour to 8 hours. Real-time PCR revealed that miR-145 expression was down-regulated in NRVMs treated with H_2_O_2_. (C), western blot demonstrated that p53 protein levels were increased at 15 min and 30 min after treatment with H_2_O_2_, but decreased afterwards. Data are presented as means ± SEM. Significance is indicated as *p<0.05 and **p<0.01, as determined by student’s t test.

### Primary Culture of Neonatal Rat Ventricular Myocytes (NRVMs)

We obtained ventricles from 1-day-old Sprague-Dawley rats and isolated cardiac myocytes through digestion with trypsin-EDTA and type 2 collagenase as previously described [19]. The protocol was approved by the Institutional Animal Care and Use Committee of the affiliated Drum Tower Hospital of Nanjing University Medical School. Briefly, the tissues were cut into small pieces and digested by 0.25% trypsin at 4°C overnight. Collagenase (Worthington, Lakewood, USA, 1 mg/ml in HBSS) was used to further digest tissues in shaking bath at 37°C for 20 min. The cell suspension was centrifuged at 1,000 rpm for 5 min and resuspended in 10% fetal bovine serum (FBS) (GIBCO) DMEM with 1 g/L glucose (GIBCO) and 10% FBS. Cells were cultured for 2 hours to allow fibroblast cells to attach to the flask. NRVMs were collected from the supernatants and cultured with DMEM containing 1 g/L glucose plus 10% FBS and 1% penicillin/streptomycin (GIBCO). Adenoviruses were used to transduce cells at indicated multiplicity of infection (MOI). Cells were further transfected with plasmid constructs 24 hours after adenovirus transduction and stimulated with various concentrations of H_2_O_2_ (Sigma, St. Louis, USA) from 48 hours later, for the indicated time courses.

### Generation of miR-145 Adenovirus

The miR-145 adenovirus used in this study contained human miR-145 gene (has-miR-145), whose mature miRNA sequence is identical to that of rat miR-145 (rno-miR-145). Adenoviruses harboring a 441–base pair DNA fragment encompassing the hsa-mir-145 gene (Ad-miR-145) were generated using the AdMax (Microbix) systems according to the manufacturers’ recommendations. An adenovirus bearing LacZ (Ad-LacZ) was obtained from Clontech. Viruses were packaged and amplified in HEK293 cells and purified using CsCl banding, followed by dialysis against 10 mmol/L Tris-buffered saline with 10% glycerol. Titering was performed on HEK293 cells using the Adeno-X Rapid Titer kit (BD Biosciences Clontech, Palo Alto, CA, USA) according to the manufacturer’s instructions.

**Figure 2:**
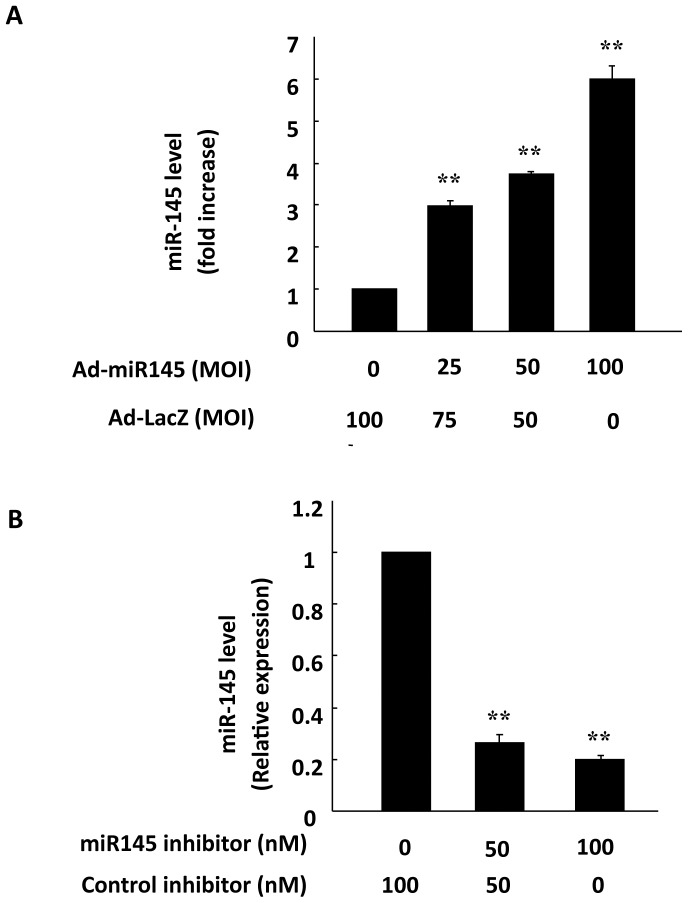
Expression of miR-145 in cardiomyocytes either transduced with Ad-miR145 and transfected with miR-145 inhibitor. Quantitative real time PCR revealed that the expression levels of miR-145 were increased after Ad-miR145 transduction (25–100 MOIs) (A), but suppressed by miR-145 inhibitor in a dose-dependent manner (50–100 nM) (B) in cultured NRVMs. Data are presented as means ± SEM. Significance is indicated as *P<0.05 and **P<0.01, as determined by student’s t test.

**Figure 3:**
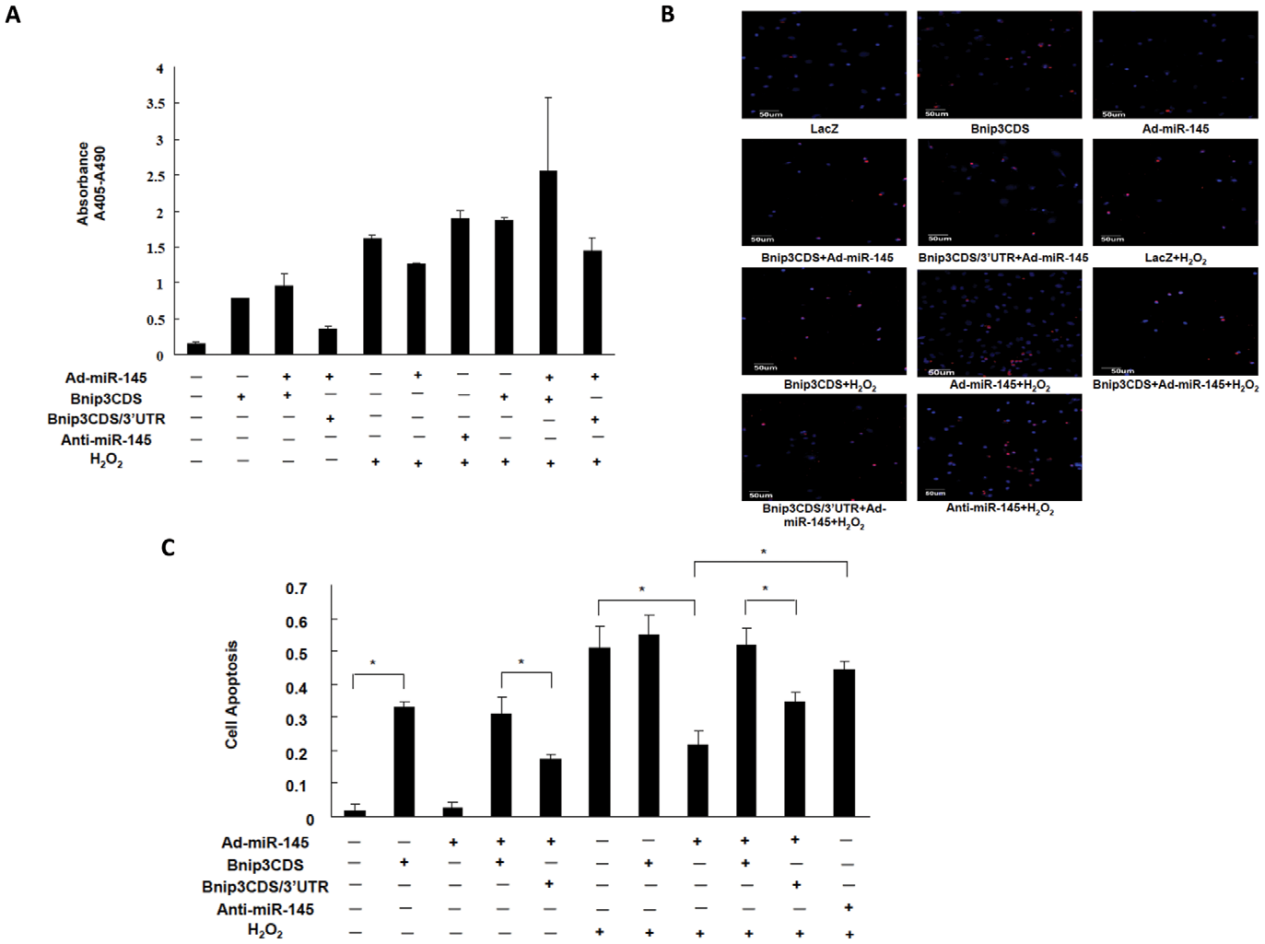
MiR-145 inhibited H_2_O_2_-induced apoptosis in cultured NRVMs. For both DNA ladder ELISA and TUNEL assay, cultured NRVMs were transduced with Ad-LacZ or Ad-miR-145 (MOI = 100) and transfected with either 0.5 µg Bnip3 CDS or Bnip3 CDS/3′UTR or rAd-psilence empty vector per 10^6^ cells; and then transfected with 10 nM miR-145 inhibitor or control inhibitor per 10^6^ cells. Cells were treated with vehicle or 50 µM H_2_O_2_ for 2 hours. In DNA ladder ELISA (A), apoptosis of cardiomyocytes in different group was evaluated by calculating A405 nm-A490 nm values. The result shows that miR-145 protected against H_2_O_2_-induced cardiomyocytes apoptosis, which was significantly enhanced by Bnip3. In TUNEL staining assay (B), the percentage of cell apoptosis was calculated by the following formula: number of TUNEL (red)-positive cells/number of DAPI (blue)-positive cells. The rates were calculated in 10 randomly chosen fields from each group. Scale bar indicates 50 µm. C, the quantitative analysis of cardiomyocyte apoptosis was determined by TUNEL staining assay. Data are presented as means ± SEM. Significance is indicated as *P<0.05 and **P<0.01, as determined by student’s t test.

### Generation of Bnip3-CDS and Bnip3-CDS-3′UTR Constructs and Cardiomyocyte Transfection

Plasmid constructs containing the coding sequence (CDS) or the coding sequence combined with the 3′UTR of rat Bnip3 (GenBank, accession No. AF243515) were prepared by cloning the indicated sequences into rAd-psilence-CMV vector (Applied Biosystems, Carlsbad, USA). Namely, Bnip3-CDS contained 621 bp from +1 to +621 nt, and Bnip3-CDS-3′UTR contained 1378 bp from +1 to +1378 nt (+1 designates the translation starting site of rat Bnip3), including the two putative binding sites for miR-145 which located in +1267∼+1272 nt and +1308∼+1313 nt respectively. Both plasmid constructs were used to transfect NRVMs using Effectene Transfection Reagent (Qiagen, Cat.No. 301425). The transfection efficiency was evaluated by transfecting eGFP-C1 empty vector (Clontech, Mountain View, CA) into NRVMs and observing the fluorescence of GFP under fluorescence microscopy (Leica DM2500-3HF-FL, Solms, Germany). The transfection efficiency reached ∼40%. MiR-145 inhibitor (rno-miR-145 inhibitor, Cat. No. miR2000437, RiBo Bio, Guangzhou, China), was transfected using Lipofectamine 2000 (Invitrogen, Carlsbad, CA, USA) when indicated.

### Luciferase Reporter Assay

Based on the rat Bnip3 mRNA sequence deposited in the GenBank database (accession No. AF243515), the fire luciferase cDNA fused with rat Bnip3 mRNA 3′UTR containing the two seed sequences for miR-145 (308 bp, +1012 to +1319 nt) and 3′UTR without the seed sequence (198 bp, +1012 to +1209 nt) were amplified separately from the genomic DNA of neonatal rat ventricle myocytes (NRVM) and cloned into the pGL3-promoter luciferase reporter vector (Promega, Madison, USA). Preconfluent H9c2 cells, in 12-well plates, were transduced with Ad-LacZ and Ad-miR-145 at indicated MOIs for 24 h, then cells were transfected with 300 ng of firefly luciferase reporter plasmid (pGL3-Luc-Bnip3 -3′UTR or pGL3-Luc-Bnip3 -3′UTR MU) and 20 ng of Renilla luciferase reporter plasmid pRL-RSV (Promega) using Lipofectamine 2000 transfection reagent (Invitrogen). Cell lysates were assayed for luciferase activity 48 h after transfection using the Luciferase Assay System (Promega) according to the instructions from the manufacturer. Firefly luciferase activity was normalized for transfection efficiency by the corresponding Renilla luciferase activity. All transfection experiments were performed at least 5 times in duplicate.

**Figure 4:**
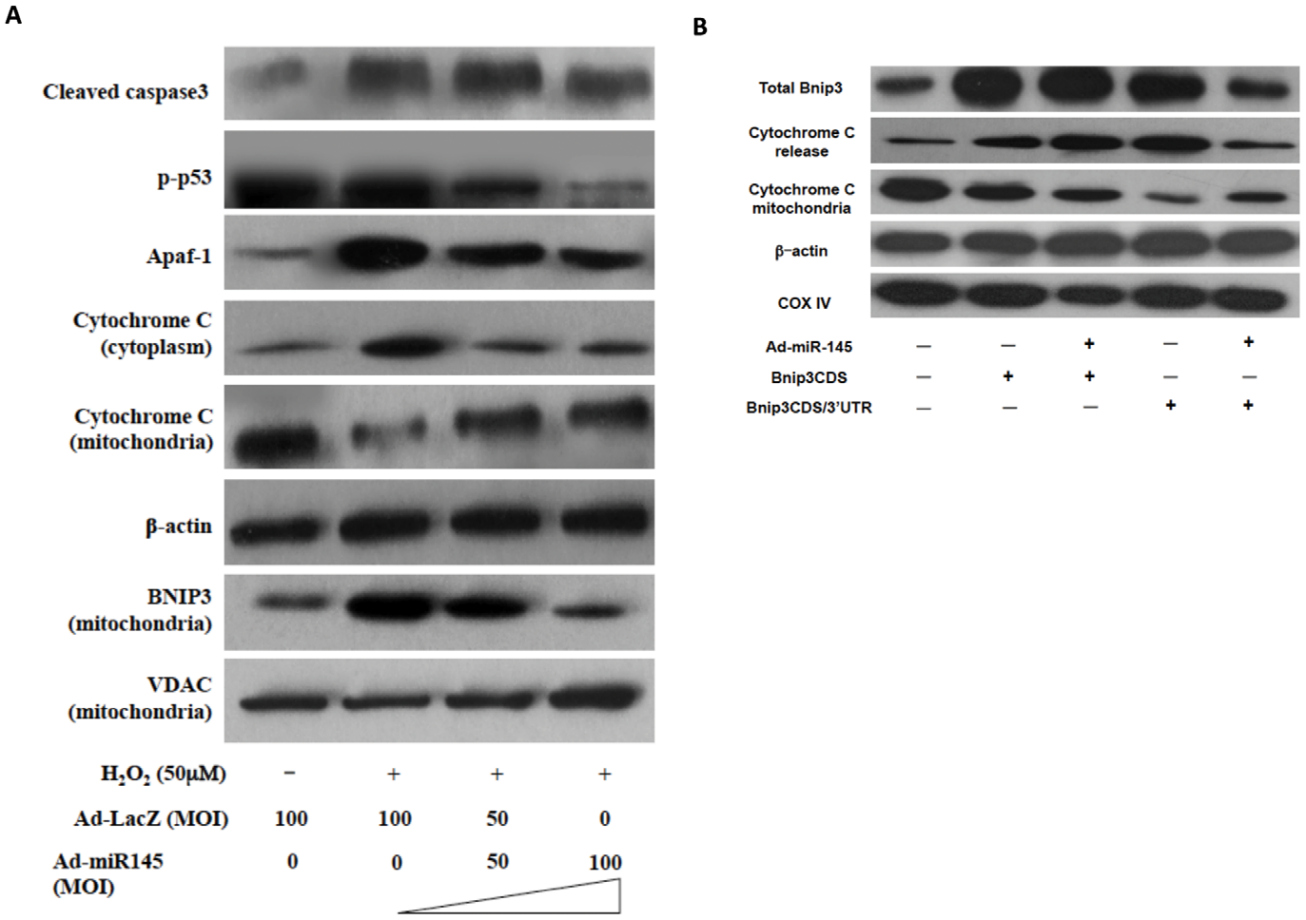
MiR-145 modulated the expression levels of key apoptosis mediators. (A), The levels of key apoptosis mediators in mitochondria apoptosis pathway, including cleaved caspase3, phospho-p53 (S15), Apaf-1, mitochondrial Bnip3, cytochrome C in mitochondria and cytoplasm, were determined by western blotting analyses. β-actin and VDAC were used as loading controls for cytoplasmic protein and mitochondrial protein detections respectively. The result shows that miR-145 over-expression inhibited the activation of mitochondrial apoptotic pathway. (B), cultured NRVMs were transduced with either Ad-miR-145 or Ad-LacZ (MOI = 100) and then transfected with either 1 µg Bnip3 CDS or Bnip3 CDS/3′UTR or rAd-psilence empty vector per 10^6^ cells. 48 hr after transfection, cells were then transfected with 20 nM either miR-145 inhibitor or control inhibitor per 10^6^ cells. Protein levels of Bnip3 (total Bnip3), cytochrome C in mitochondria, and cytoplasm (cytochrome C release) were detected by western blotting. β-actin and COX IV served as loading controls for cytoplasmic and mitochondrial proteins, respectively. The result shows that miR-145 protected against Bnip3-induced cytochrome C release. All results were repeated in three independent experiments.

### Quantitative Real-time PCR

Total RNA was extracted from cultured cells using Trizol reagent (Invitrogen, Carlsbad, CA, USA) following the manufacturer’s instructions. Two micrograms of total RNA were reverse transcribed in a total volume of 20 µl, and real-time PCR using SYBR green fluorescence was performed (BIO-RAD) to detect miR-145 expression as previously described [20]. Complementary DNA was synthesized using a miR-145-specific stem-loop primer: 5′-CTCAACTGGTGTCGTGGAGTCGGCAATTCAGTTGAGAGGGATTC-3′. Quantitative polymerase chain reaction used the following primers: forward, 5′- CGCGCTCGAGCCCAGAGCAATAAGCCACAT -3′; reverse, 5′-GGTGTCGTGGAGTCGGCAATTCAGTTGAG-3′. The primers for small nuclear RNA U6 (U6) were: forward, 5′-CTCGCTTCGGCAGCACA-3′; reverse, 5′-AACGCTTCACGAATTTGCGT-3′. The reverse primer of U6 was also used in reverse transcription of U6. PCR condition was 95°C for 15 min, followed by 40 cycles at 95°C for 15 sec and 60°C for 1 min. Samples were run in duplicate with RNA preparations from three independent experiments. Each real-time PCR reaction consisted of 2 µl RT product, 10 µl SYBR Green PCR Master Mix, and 500 nM forward and reverse primers. Reactions were carried out on a MyiQ Single Color Real-time PCR Detection System (Bio-Rad) The fold change in expression of each gene was calculated using the 2^−△△CT^ method with U6 as an internal control.

**Figure 5:**
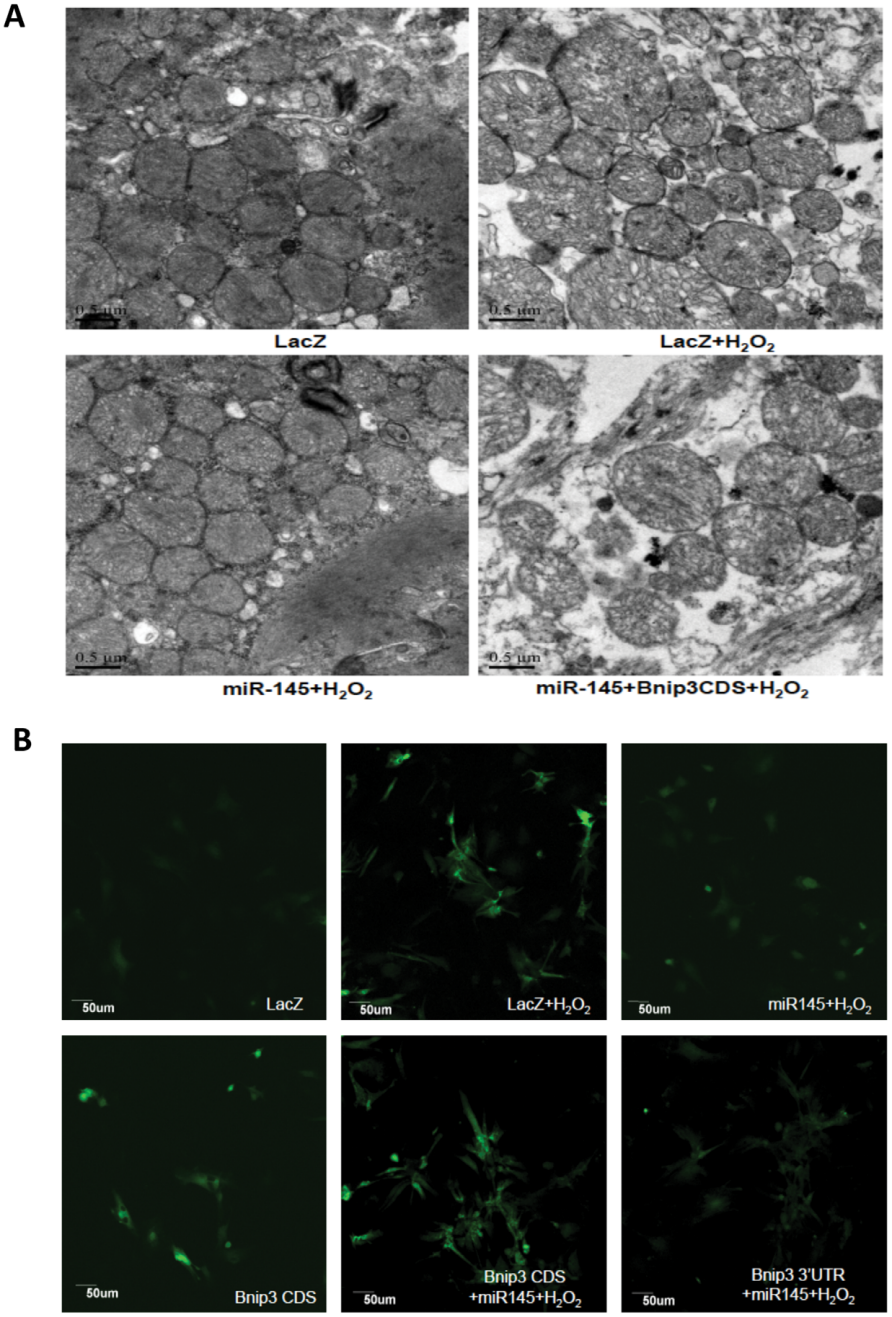
MiR-145 protected against H2O2-induced mitochondrial structural disruption and decreased the ROS production. For both electron transmission microscopy and ROS staining, NRVMs were transduced with Ad-miR-145 or Ad-LacZ (MOI = 100) and transfected with either 1 µg Bnip3 CDS or Bnip3 CDS/3′UTR or rAd-psilence empty vector per 10^6^ cells, and then treated with 50 µM H_2_O_2_ for 2 h. (A), under electron transmission microscopy, it was shown that miR-145 attenuated H_2_O_2_-induced mitochondrial structural disruption (swelling, rupture and loss of cristae). Forced expression of Bnip3 CDS enhanced mitochondrial disruption and abrogated the protective effect of miR-145. Scale bars indicate 0.5 µm. (B), ROS staining was performed using DCFH-DA, and was observed with confocal microscopy. The panels show that miR-145 reduced H_2_O_2_-triggered ROS production, which was promoted by Bnip3. The result represents the study of three independent experiments.

### Western Blotting Analysis

Cardiomyocytes were transduced with Ad-miR145 or Ad-LacZ at 50 or 100 MOI and treated with 50 µM H_2_O_2_ or vehicle as indicated. The whole lysates were used to detect cleaved caspase 3, phosphorylated p53 (p-p53, Ser-15), Bnip3, Apaf-1, and β-actin. Cytochrome C release was evaluated by detecting cytochrome C in cytoplasmic and mitochondrial lysates. The isolated mitochondrial lysates were also used to detect Bnip3, COX IV or VDAC. The primary antibodies were as follows: cytochrome C, Bnip3, p-p53 (Ser-15), Apaf-1, COX IV and Cleaved Caspase 3 (Asp-175), all of which were rabbit polyclonal antibodies from Bioworld Corporation (Dublin, USA); anti-VDAC rabbit polyclonal antibody from Cell signaling Technology Inc (Danvers, USA) and anti-β-actin mouse monoclonal antibody from Bioworld Corporation (Dublin, USA).

### TUNEL Assay

For TUNEL staining, cells were cultured in chambers (Milipore, Billerica, USA) at a density of 10^5^ cells/chamber and transduced with Ad-miR-145 or Ad-LacZ. The miR-145 inhibitor along with control inhibitor was transfected 24 hours after adenoviruses transduction. 48 hours after transduction, 50 µM H_2_O_2_ were added to the cells for 2 hours. After treatment, cells were fixed by 4% paraformaldehyde and TUNEL staining was performed using the In Situ Cell Death Detection Kit (Roche, Mannheim, Germany) according to the manufacturer’s instructions.

### ELISA for DNA Ladder Detection

DNA ladder detection was performed according to the instruction of Cell Death Detection ELISAPlus (Roche). Briefly, cells cultured in 12-well plate were washed twice with PBS to remove the necrosis. Lysis buffer was used to lyse the cells for 30 min at room temperature, followed by incubation with the immunoreagent containing anti-histone-biotin and anti-DNA-POD for 2 hours with gentle shaking. Substrate buffer (ABTS) was added to develop the color and the results were presented as absorbance A405–A490.

### DCFH-DA Staining

2′,7′-Dichlorodihydrofluorescein diacetate (DCFH-DA) dye (Sigma) was diluted with DMEM medium and added to the living cells cultured in chambers. Cells were then incubated in 5% CO_2_ at 37°C for 50 min and washed twice with PBS. Chambers were subject to confocal microscopy examination.

### Electron Transmission Microscopy

Cells were harvested by a gentle digestion with 0.25% trypsin and fixed with 2% glutaraldehyde. Osmium tetroxide (1% in 0.1 mol/L cacodylate) was used for postfixation. Copper grids were stained with 2% uranyl acetate followed by dehydration with series of ethanol. Samples were embedded with epoxy resin and polymerized at 60°C. The images were acquired from electron transmission microscopy (JEOL JEM-1400, Tokyo, Japan) scan.

### Statistics

All data were presented as the means±SE from at least three independent experiments. Statistical analysis was performed by Student’s t test, using SPSS software. Statistic significance was demonstrated as *, or **, with * indicating p<0.05, and ** indicating p<0.01.

**Figure 6:**
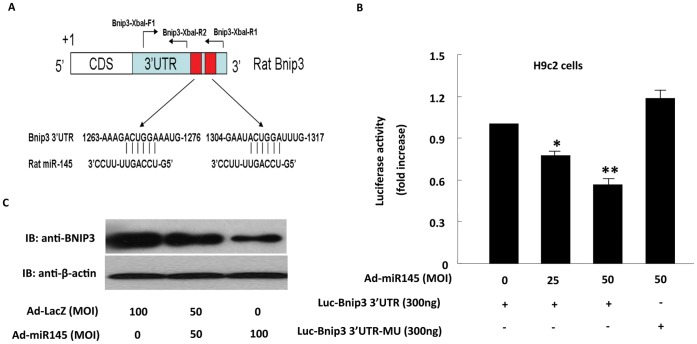
MiR-145 suppressed expression of Bnip3 through binding to its 3′UTR. (A), a representative illustration of the putative binding sites for miR-145 in rat Bnip3 3′-UTR. (B), miR-145 targets two seed sequences (+1267∼+1272 nt and +1308∼+1313 nt) of Bnip3 3′-UTR. Luciferase assay was performed in H9c2 cells using pGL3 reporter vector fused with either Bnip3 wild-type 3′-UTR or Bnip3 3′-UTR mutant. Over-expression of miR-145 significantly decreased the activity of luciferase gene fused with Bnip3 wild-type 3′-UTR, but had no effect on the activity of luciferase fused with Bnip3 3′-UTR mutant; (C), Detection of Bnip3 expression by western blot in the whole lysates of NVRMs transduced with different dosages of Ad-miR-145 (50 and 100 MOI). The result showed that the protein level of Bnip3 was suppressed by miR-145 in a dose-dependent manner. Data are presented as means ± SEM. Significance is indicated as *P<0.05 and **P<0.01, as determined by student’s t test.

## Results

### Expression of MiR-145 was Down-regulated in the Heart in Response to I/R Injury

To investigate whether miR-145 expression is altered during myocardial I/R injury, Stem-loop real-time PCR was used to detect the miR-145 mRNA levels in ischemia/reperfused myocardial tissues. As shown in figure 1A, the expression level of miR-145 was significantly decreased, by approximately 50%, in reperfused myocardial tissues. Likewise, in cultured cardiomyocytes treated with 50 µM H_2_O_2_ for 0.5 hour to 8 hours, the miR-145 mRNA levels were significantly down-regulated (figure 1B). Since previous studies suggest a role of p53 in regulating miR-145 expression [21], we further detected the p53 expression in H_2_O_2_-treatment cardiomyocytes through western blot and found that the protein levels of p53 increased in the first 30 min following H_2_O_2_ treatment, but decreased afterwards (figure 1C).

### Over-expressing miR-145 Attenuated H_2_O_2_-induced Apoptosis in Cardiomyocytes

To determine whether miR-145 plays a role in the regulation of H_2_O_2_-induced apoptosis in cardiomyocytes, we performed the adenovirus-mediated over-expression of miR-145. Quantitative real-time PCR was performed to evaluate the miR-145 levels in cultured cardiomyocytes either transduced with Ad-miR145 or transfected with miR-145 inhibitor. In fact, cardiomyocytes express a high level of endogenous miR-145 (Ct value ∼12, data not shown) and transduction of cardiomyocytes with Ad-miR145 further increased the level of miR-145 in a dose-dependent manner (figure 2A). Transfection of miR-145 inhibitor significantly suppressed the miR-145 expression (figure 2B). Noticeably, over-expression of miR-145 substantially inhibited the H_2_O_2_ induced cardiomyocyte apoptosis, as determined by TUNEL staining and DNA ladder ELISA, whereas transfection of miR-145 inhibitor increased the rate of H_2_O_2_-induced cardiomyocyte apoptosis (figure 3A–C). Furthermore, the anti-apoptotic effect of miR-145 was neutralized when Bnip3, one of the predicted miR-145 targets, was over-expressed. In both DNA ladder ELISA and TUNEL assay, over-expression of either Bnip3 CDS (containing only coding sequence of Bnip3) or Bnip3 CDS/3′UTR (containing coding sequence combined with the 3′UTR of Bnip3) resulted in an increased apoptosis rate of cardiomyocytes, in both normal and oxidative context. MiR-145 significantly lowered the apoptosis rate in the existence of Bnip3 CDS/3′UTR, but had mild effect in the existence of Bnip3 CDS (figure 3A–C). Taking together, these results suggest that miR-145 plays a protective role against the oxidative stress-induced cardiomyocyte apoptosis and this regulation of miR-145 is linked with Bnip3. Since Bnip3 is directly involved in the mitochondrial apoptotic pathway, we asked whether miR-145 could regulate the mitochondrial apoptotic pathway. We thus performed western blot to detect the expression levels of key molecules in the mitochondrial apoptotic pathway, such as cleaved caspase 3, Apaf-1, phosphorylated p53 (S15), cytochrome C, and mitochondrial Bnip3. Indeed, H_2_O_2_ treatment markedly increased the levels of cleaved caspase 3, which were only slightly decreased by miR-145 over-expression. The levels of Apaf-1, cytoplasmic cytochrome C, and mitochondrial Bnip3 were significantly increased in cells treated with H_2_O_2_, but markedly decreased by over-expression of miR-145 in a dose-dependent manner (figure 4A). Consistent with apoptosis detection assays, over-expression of either Bnip3 CDS or Bnip3 CDS/3′UTR triggered cytochrome C release, whereas over-expression of miR-145 suppressed the Bnip3 CDS/3′UTR-induced cytochrome C translocation (figure 4B). These results support that miR-145 regulated cardiomyocyte apoptosis through modulating the mitochondrial apoptotic pathway.

### MiR-145 Protected Against Mitochondrial Structure Disruption and Inhibited ROS Production

Since the activation of the mitochondrial apoptotic pathway in cardiomyocytes typically causes the structural changes and dysfunction of mitochondria [1] [4], we attempted to investigate the effect of miR-145 upon mitochondrial morphology. Treatment of the Ad-LacZ transduced cells with 50 µM H_2_O_2_ for 2 hours led to a massive swelling of mitochondria and loss of cristae, as determined by electron transmission microscopy (figure 5A). In contrast, in miR-145-over-expressing cardiomyocytes, although small vesicles were presented in mitochondria, the outer membranes of mitochondria were largely intact, the cristae were evident, and the organization of mitochondria was neat and regular (figure 5A). We also detected the overall ROS generation by using a ROS-sensitive dye 2′,7′-dichloruorescein-diacetate (DCFH-DA) [22]. As shown in Figure 5C, H_2_O_2_ and Bnip3 CDS, separately or combinatorially led to a strong staining of DCFH-DA, whereas miR-145 produced a relatively dim DCFH-DA staining in cardiomyocytes treated with H_2_O_2_ and co-expressing Bnip3 CDS/3′UTR (figure 5B), suggesting that miR-145 attenuated the production of ROS and Bnip3 might be at its downstream.

### MiR-145 Regulated Bnip3 Expression through Targeting its 3’UTR

To examine whether Bnip3 is a direct target of miR-145, we resorted to luciferase reporter assay. Before, it has been reported that human Bnip3 was a direct target of miR-145 [15]. Sequence analysis of rat BNIP-3′UTR revealed two putative miR-145 binding sites located at 1267–1272 nt and 1308–1313 nt, which is highly conserved in both Homo sapiens and Rattus norvegicus (figure 6A). Accordingly, we constructed two reporter plasmids by cloning the rat Bnip3 3′UTR containing or without the two miR-145 putative binding sites (1267–1272 nt and 1308–1313 nt) (designated as WT and mutant, respectively) into the 3′UTR of pGL3 vector. Indeed, over-expression of miR-145 markedly down-regulated the activity of luciferease gene fused with the Bnip3 WT-3′-UTR (luc-Bnip3 3′UTR). In contrast, over-expression of miR-145 barely affected the activity of luciferase gene fused with the Bnip3 3′-UTR mutant (luc-Bnip3 3′UTR-MU) (figure 6B). The protein level of Bnip3 also saw a marked dose-dependent down-regulation when Ad-miR-145 was transduced at different MOIs (0, 50, and 100) (Figure 6C). Taking together, these results suggest that Bnip3 is a direct target of miR-145 in cardiomyocytes. MiR-145 regulated Bnip3 expression through targeting the specific binding cites in the 3′UTR of Bnip3.

## Discussion

Clinically, myocardial ischemia-reperfusion is an acute and severe injury and the extensive apoptosis and necrosis of cardiomyocytes at the earliest stage of reperfusion account for most of the clinical manifestations [1]. At cellular level, oxidative stress, calcium overload and ATP depletion are three essential pathological features of the reperfused heart cells and are closely connected with the activation of the mitochondrial apoptotic pathways [23]. Recently, an emerging role of microRNAs in regulating mitochondrial functions has been explored in cardiomyocytes [24] [20]. Here, we demonstrate that miR-145 protects against the activation of mitochondria apoptotic pathway in cardiomyocytes under oxidative stress through directly targeting mitochondrial membrane protein Bnip3.

MiR-145 was considered to be a tumor suppressor microRNA and was shown to suppress tumor cell proliferation and induce cell apoptosis in a variety of tumor cell lines [7]–[9] [25]. The functional significance of miR-145 in the heart, however, has not been explored thus far. In the present study, we demonstrated that miR-145 exerted a potent protective effect against the oxidative stress-induced apoptosis in cardiomyocytes (figure 3A–C). Previously, miR-145 has been shown to inhibit tumor growth mostly through targeting pro-proliferative molecules such as p70S6K and c-myc [25]–[26]. However, in cardiomyocytes, we found that miR-145 modulated the mitochondrial pathway by directly targeting key intermediates in the mitochondrial apoptosis machinery (figure 4, 6C). Indeed, the expression of miR-145 has been shown to be down-regulated in a variety of cancer cells as we observed here in cardiomyocytes treated with H_2_O_2_ (figure 1B). However, this down-regulation may result in different cellular behaviors, since most cancer cells, unlike cardiac myocytes, are far less sensitive to the toxicity of ROS and the pathological mPTP opening [27]. The down-regulation of miR-145 was also noted in vascular smooth muscle cells treated with H_2_O_2_ [10]. Nevertheless, the mechanism of the down-regulation of miR-145 by oxidative stress is unclear. p53 has been identified to positively regulate miR-145 maturation [28]. In tumor cells, the p53 mutation and hypermethylation of miR-145 promoter seem to primarily account for the miR-145 down-regulation [21]. In our study, p53 was found to be transiently up-regulated at 15 min and 30 min following the treatment of H_2_O_2_, but was subsequently decreased at 1 hour and 2 hour (figure 1C), while miR-145 was continuously down-regulated from 30 min to 8 h after H_2_O_2_ treatment, indicating that besides p53, alternative mechanisms may be involved in regulating the miR-145 expression in cardiomyocytes. Thus, it would be interesting to investigate whether a change of methylation status in miR-145 promoter or an aberrant processing of pre-miR145 may account for the miR-145 down-regulation in cardiomyocytes in response to oxidative stress.

In the present study, we identified Bnip3 as a direct target of miR-145 in rat cardiomyocytes. Indeed, ectopic expression of Bnip3 significantly induced apoptosis in cardiomyocytes and this was partially inhibited when miR-145 was over-expressed. These results show that the protection of miR-145 against cardiomyocytes oxidative stress is, at least in part, functionally attributed to its suppression of Bnip3. It is interesting that Bnip3 has recently been shown to transduce the apoptotic signal in a caspase-independent manner [15] [29]. Conventionally, the mitochondrial apoptotic pathway constitutes of the release of mitochondrial proteins such as cytochrome C into cytoplasm, followed by the activation of caspase proteins. However, in the adult heart, the expression levels of caspase proteins are low and the caspase-dependent apoptosis machinery is largely silenced [15]. Indeed, our findings support this viewpoint by showing that the protein levels of released cytochrome C and Apaf-1 were markedly decreased by miR-145 over-expression, whereas the protein levels of cleaved caspase3 were only moderately decreased. Considering that Apaf-1 functions as a linker between cytochrome C and caspase proteins [30], our results suggest there exist non-caspase signaling pathways linking cytochrome C and Apaf-1 to the DNA degradation and miR-145-mediated protection against cardiac apoptosis may be through these alternative pathways. Coincidently, in urothelial cancer cell line, miR-145 also induces a caspase3-independent cell death [8], in a similar way to the caspase-independent apoptosis mediated by Bnip3 [15]. Together, these data support an essential role of miR-145 in the regulation of the mitochondrial machinery through modulating the levels of molecules upstream of caspase activation [8].

The increased ROS level is a hallmark in oxidative stress induced cardiomyocyte apoptosis. Strategies to interrupt the cycle of ROS production may be hopeful in apoptosis salvage [31]. Excessive amount of ROS not only alters the redox environment in cytoplasm and disrupts mitochondrial membrane, but also facilitates Bnip3 activation by allowing Bnip3 to insert into the mitochondrial membrane. Strikingly, we demonstrated that miR-145 over-expression markedly attenuated the production of ROS in cardiomyoctes in response to oxidative stress, suggesting that miR-145 may exert the cardioprotective effects through regulating not only the expression of Bnip3, but also the activation of it.

In summary, we have demonstrated that the expression of miR-145 is substantially down-regulated in both ischemia/reperfused heart and H_2_O_2_-treated cardiomyocytes and that miR-145 over-expression confers cardiac protection against oxidative stress-induced cardiomyocyte apoptosis through directly inhibiting the Bnip3 expression and the ROS generation under oxidative stress conditions. Since oxidative stress is a common pathological factor shared by many cardiovascular diseases, such as cardiac hypertrophy, cardiomyopathy, and acute myocardial infarction [31]–[32], identification of miR-145 as a novel cardioprotective molecule may potentially enable us to develop additional therapeutic strategies for prevention and treatment of cardiovascular disease.
